# Clinical impact of serum bilirubin levels on kidney transplant outcomes

**DOI:** 10.1038/s41598-021-86330-y

**Published:** 2021-03-25

**Authors:** Juhan Lee, Eun Jin Kim, Jae Geun Lee, Beom Seok Kim, Kyu Ha Huh, Myoung Soo Kim, Soon Il Kim, Yu Seun Kim, Dong Jin Joo

**Affiliations:** 1 Department of Surgery, Yonsei University College of Medicine, Seoul, Republic of Korea; 2 Department of Internal Medicine, Yonsei University College of Medicine, Seoul, Republic of Korea

**Keywords:** Biomarkers, Nephrology, Urology

## Abstract

Serum bilirubin, a potent endogenous antioxidant, has been associated with decreased risks of cardiovascular disease, diabetes, and kidney disease. However, the effects of serum bilirubin on kidney transplant outcomes remain undetermined. We analyzed 1628 patients who underwent kidney transplantations between 2003 and 2017. Patients were grouped into sex-specific quartiles according to mean serum bilirubin levels, 3–12 months post-transplantation. Median bilirubin levels were 0.66 mg/dL in males and 0.60 mg/dL in females. The intra-individual variability of serum bilirubin levels was low (9%). Serum bilirubin levels were inversely associated with graft loss, death-censored graft failure, and all-cause mortality, independent of renal function, donor status, and transplant characteristics. Multivariable analysis revealed that the lowest serum bilirubin quartile was associated with increased risk of graft loss (HR 2.64, 95% CI 1.67–4.18, *P* < 0.001), death-censored graft failure (HR 2.97, 95% CI 1.63–5.42, *P* < 0.001), and all-cause mortality (HR 2.07, 95% CI 1.01–4.22, *P* = 0.046). Patients with lower serum bilirubin were also at greater risk of rejection and exhibited consistently lower glomerular filtration rates than those with higher serum bilirubin. Serum bilirubin levels were significantly associated with transplantation outcomes, suggesting that bilirubin could represent a therapeutic target for improving long-term transplant outcomes.

## Introduction

Kidney transplantation is the treatment of choice for most patients with end-stage renal disease. Despite considerable advances in short-term outcomes, long-term kidney transplantation outcomes remain suboptimal^1,2^. The causes associated with late graft failure are complex and multifactorial. Although immunologic risks are important, non-immunologic factors, including cardiovascular disease, metabolic disturbances, the recurrence of native kidney disease, and endothelial dysfunction have also been associated with late graft failure^3,4^. Therefore, the identification of a useful monitoring tool or therapeutic target for standard clinical practice, beyond the evaluation of graft function, donor-specific antibodies, and histologic findings, has been difficult^5,6^.

Bilirubin has long been viewed as a useless metabolite produced by heme catabolism, with little or no physiological function, but with potential toxicity. However, accumulating clinical observations have indicated that circulating bilirubin, at levels within the physiological range, may protect against cardiovascular disease, diabetes, and all-cause mortality^7–9^. These findings have also been extended to protection against kidney disease progression^10,11^. These beneficial effects have largely been attributed to the antioxidant property of bilirubin^12,13^. Recent studies have further suggested that bilirubin may exert anti-inflammatory, immunomodulatory, and endothelial protective effects^14^.

Despite the beneficial effects associated with circulating bilirubin levels, whether serum bilirubin levels affect long-term graft outcomes after kidney transplantation remains unclear^15^. Therefore, we examined the association between serum bilirubin levels and graft and patient survival, graft function, and rejection in a large cohort of kidney transplant recipients.

## Results

### Baseline characteristics

**Figure 1:**
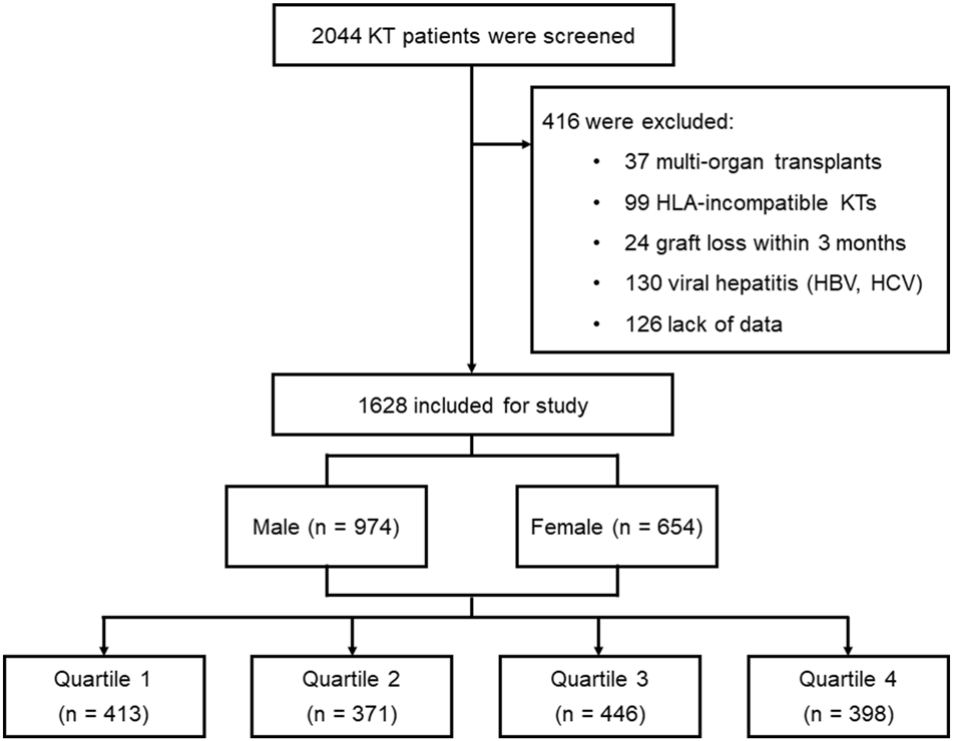
Study flow chart.

**Figure 2:**
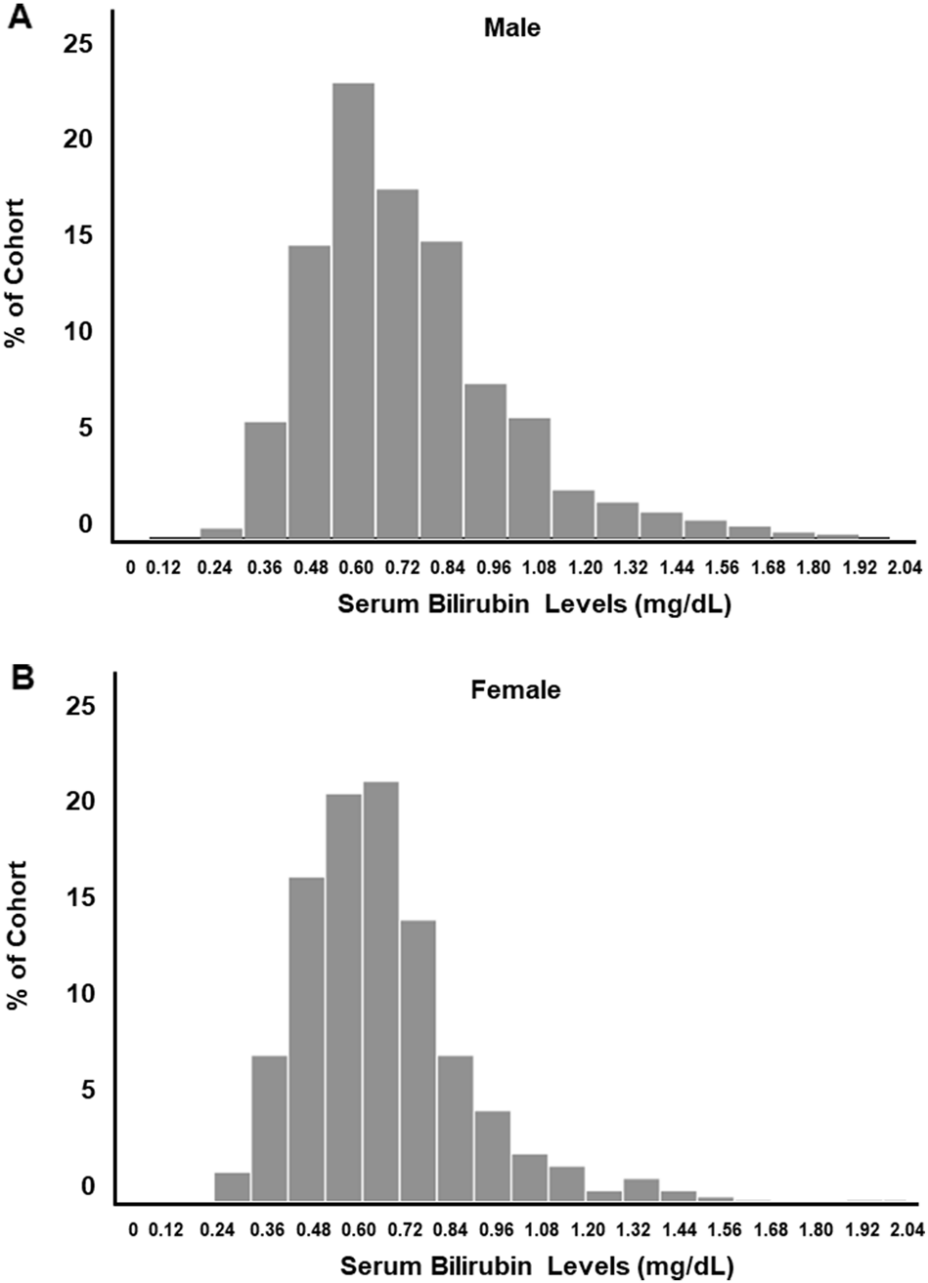
Distribution of serum bilirubin levels by sex. (**A**) Male. (**B**) Female.

A total of 1628 patients were included in this study, who underwent an average of 8.6 serum bilirubin tests per patients between 3 and 12 months after transplantation (Fig. 1). The distribution of mean serum bilirubin levels, according to quartile and sex, is shown in Fig. 2. Mean serum bilirubin levels in this cohort were right-skewed, with median levels of 0.66 mg/dL (IQR 0.50–0.85) in males and 0.60 mg/dL (IQR 0.50–0.74) in females. The intra-individual variability of serum bilirubin levels was low, at 9%.

The baseline characteristics of patients according to serum bilirubin quartiles are presented in Table 1. No significant differences in age, sex, body mass index, and dialysis vintage were observed among the groups. Hepatic synthesis markers were not significantly different among groups. Patients with high serum bilirubin levels tended to receive transplants from younger donors. Patients with low serum bilirubin levels received more deceased donor kidney transplants and were more likely to develop delayed graft function. Although mean donor eGFR of the lowest bilirubin group was significantly lower than other groups, mean donor eGFR of the group was greater than 90 mL/min/1.73m^2^. The median follow-up duration was 87 months (IQR 51.3–127.0).

**Table 1:** Baseline characteristics of patients, stratified by sex-specific serum bilirubin levels.

	Quartile 1 (N = 413)	Quartile 2 (N = 371)	Quartile 3 (N = 446)	Quartile 4 (N = 398)	*P*
Female, *n* (%)	163 (39.5)	134 (36.1)	194 (43.5)	163 (41.0)	0.189
Age, years	44.4 ± 12.4	44.7 ± 11.8	45.4 ± 11.3	45.9 ± 11.1	0.235
Mean level of bilirubin, mg/dL	0.4 ± 0.1	0.6 ± 0.1	0.7 ± 0.1	1.1 ± 0.3	< 0.001
Body mass index, kg/m^2^	22.7 ± 3.2	22.6 ± 3.5	22.2 ± 3.1	22.4 ± 3.3	0.232
HLA mismatch number	2.9 ± 1.5	3.0 ± 1.5	2.9 ± 1.5	2.7 ± 1.5	0.083
Re-transplant, *n* (%)	36 (8.7)	32 (8.6)	35 (7.8)	28 (7.0)	0.54
Dialysis vintage, months	39.5 ± 50.1	37.0 ± 48.3	34.7 ± 46.4	38.3 ± 54.5	0.563
Hepatic synthesis marker					
Albumin, g/dL	4.3 ± 0.4	4.4 ± 0.4	4.4 ± 0.3	4.4 ± 0.3	0.377
Prothrombin time, INR	0.9 ± 0.1	0.9 ± 0.1	1.0 ± 0.1	1.0 ± 0.2	0.301
Female donor, *n* (%)	205 (49.6)	197 (53.1)	212 (47.5)	202 (50.8)	0.455
Donor age, years	44.4 ± 12.9	42.4 ± 12.0	41.3 ± 12.3	40.3 ± 12.0	< 0.001
Donor eGFR, ml/min/1.73m^2^	91.5 ± 29.4	95.7 ± 29.7	98.7 ± 26.5	101.2 ± 28.5	< 0.001
Deceased donor, *n* (%)	140 (33.9)	108 (29.1)	99 (22.2)	89 (22.4)	< 0.001
Cause of death, *n* (%)					0.736
Hypoxia	31 (22.1)	21 (19.4)	27 (27.3)	22 (24.7)	
Cerebrovascular disease	73 (52.1)	54 (50.0)	52 (52.5)	40 (44.9)	
Trauma	32 (22.9)	30 (27.8)	18 (18.2)	24 (27.0)	
CNS tumor	2 (1.4)	1 (0.9)	2 (2.0)	1 (1.1)	
Others	2 (1.4)	2 (1.9)	0	2 (2.2)	
Delayed graft function, *n* (%)	45 (10.9)	27 (7.3)	31 (7.0)	20 (5.0)	0.015
Renin-angiotensin system inhibitors	200 (48.4)	149 (40.2)	152 (34.1)	135 (33.9)	< 0.001
HMG CoA reductase inhibitors	212 (51.3)	207 (55.8)	244 (54.7)	259 (65.1)	< 0.001

Table 2 shows the immunosuppressive treatment in our cohort. No significant differences in induction regimen were observed among the groups. Tacrolimus was used more frequently in patients with low serum bilirubin levels than in those with high levels. There were significant differences in the tacrolimus doses and trough levels, whereas there were no significant differences in the cyclosporine doses and trough levels among the groups.

**Table 2:** Immunosuppression, stratified by sex-specific serum bilirubin levels.

	Quartile 1 (N = 413)	Quartile 2 (N = 371)	Quartile 3 (N = 446)	Quartile 4 (N = 398)	*P*
*Induction agent, n (%)*					
No	59 (14.3)	44 (11.9)	59 (13.2)	53 (13.3)	0.709
Basiliximab	343 (83.1)	317 (85.4)	381 (85.4)	338 (84.9)	
Anti-thymocyte globulin	11 (2.7)	10 (2.7)	6 (1.3)	7 (1.8)	
Maintenance agent, *n* (%)					0.007
Tacrolimus	337 (81.6)	280 (75.5)	333 (74.7)	284 (71.4)	
Cyclosporine	76 (18.4)	91 (24.5)	113 (25.3)	114 (24.4)	
*Tacrolimus level, ng/mL*					
1 month	6.7 ± 3.4	6.4 ± 3.4	6.3 ± 2.9	5.9 ± 3.1	0.004
3 months	6.6 ± 3.1	6.3 ± 2.7	6.3 ± 2.6	5.8 ± 2.4	0.002
6 months	6.5 ± 2.9	6.4 ± 2.8	6.2 ± 2.4	5.9 ± 2.2	0.021
12 months	6.1 ± 2.7	6.1 ± 2.6	6.2 ± 2.7	5.9 ± 2.5	0.271
*Tacrolimus dose, mg/day*					
1 month	5.8 ± 3.5	4.9 ± 3.0	4.9 ± 3.0	4.5 ± 2.7	< 0.001
3 months	5.3 ± 3.0	4.6 ± 2.5	4.7 ± 2.7	4.1 ± 2.4	< 0.001
6 months	5.0 ± 2.9	4.4 ± 2.3	4.4 ± 2.5	4.0 ± 2.3	< 0.001
12 months	4.7 ± 2.8	3.9 ± 2.1	4.0 ± 2.3	3.7 ± 2.1	< 0.001
*Cyclosporine level, ng/mL*					
1 month	161.8 ± 65.8	156.3 ± 66.7	163.5 ± 63.1	147.0 ± 51.7	0.259
3 months	120.6 ± 57.8	121.7 ± 41.3	122.8 ± 40.5	130.0 ± 47.3	0.584
6 months	98.1 ± 44.5	112.5 ± 68.2	117.7 ± 50.5	118.2 ± 56.6	0.22
12 months	101.7 ± 58.0	111.2 ± 75.0	114.7 ± 62.5	112.6 ± 46.0	0.71
*Cyclosporine dose, mg/day*					
1 month	241.4 ± 97.8	230.1 ± 88.6	242.7 ± 89.0	234.2 ± 88.6	0.74
3 months	188.8 ± 85.6	189.5 ± 69.5	202.0 ± 68.3	206.9 ± 76.3	0.247
6 months	153.1 ± 87.9	164.3 ± 73.9	168.9 ± 72.6	170.7 ± 62.8	0.198
12 months	136.7 ± 81.4	147.9 ± 62.4	147.2 ± 58.0	149.4 ± 63.0	0.093

### Risk factors associated with the lowest serum bilirubin levels

To confirm the risk factors associated with low serum bilirubin levels, we performed multivariable logistic regression analyses using the lowest serum bilirubin quartile as a binary, dependent variable. In the multivariable analysis, recipient age, tacrolimus use, renin-angiotensin system inhibitor use, and hemoglobin, total cholesterol, and high-density lipoprotein cholesterol levels at 3 months post-transplantation remained associated with low serum bilirubin levels (Table 3).

**Table 3:** Risk factors associated with the lowest serum bilirubin quartile.

Variable	Univariate		Multivariate	
	OR (95% CI)	*P*	OR (95% CI)	*P*
Age, years	0.99 (0.98–1.00)	0.136	0.97 (0.96–0.99)	< 0.001
Female	0.96 (0.77–1.21)	0.735		
Body mass index, kg/m^2^	1.02 (0.99–1.06)	0.146	1.03 (0.97–1.09)	0.397
HLA mismatch (per 1 mismatch)	1.05 (0.97–1.13)	0.203		
Deceased donor (versus living)	1.59 (1.25–2.03)	< 0.001	1.19 (0.76–1.85)	0.453
Donor age, y	1.02 (1.01–1.03)	< 0.001	1.01 (0.99–1.02)	0.444
Donor eGFR, ml/min/1.73m^2^	0.99 (0.98–0.99)	< 0.001	1.00 (0.99–1.01)	0.159
Tacrolimus (versus cyclosporine)	1.57 (1.19–2.08)	0.002	1.86 (1.12–3.09)	0.017
Renin-angiotensin system inhibitors	1.68 (1.34–2.10)	< 0.001	1.47 (1.03–2.11)	0.035
HMG CoA reductase inhibitors	0.75 (0.60–0.94)	0.012	1.00 (0.69–1.45)	0.998
Laboratory findings at 3 months				
eGFR, ml/min/1.73m^2^	0.98 (0.97–0.98)	< 0.001	0.99 (0.98–1.00)	0.141
Hemoglobin, mg/dL	0.63 (0.58–0.68)	< 0.001	0.60 (0.52–0.69)	< 0.001
Phosphorus, mg/dL	0.92 (0.76–1.11)	0.388		
Uric acid, mg/dL	1.12 (1.03–1.23)	0.008	0.91 (0.80–1.03)	0.127
Total cholesterol, mg/dL	1.00(1.00–1.01)	0.058	1.01 (1.00–1.01)	0.004
HDL cholesterol, mg/dL	0.98 (0.97–0.99)	< 0.001	0.98 (0.96–0.99)	0.001
Triglyceride, mg/dL	1.01 (1.00–1.01)	< 0.001	1.00 (1.00–1.01)	0.612

### Serum bilirubin and graft outcomes

**Figure 3:**
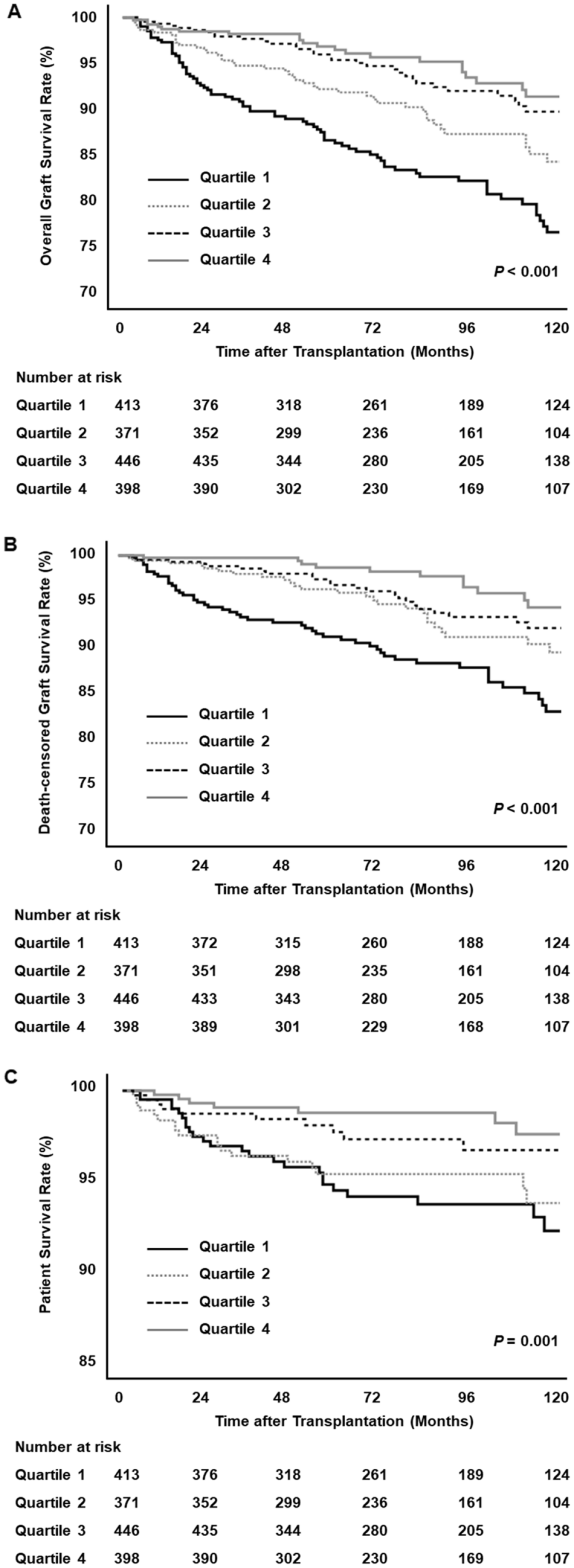
Graft and patient survival according to serum bilirubin level: (**A**) overall graft survival, (**B**) death-censored graft survival, and (**C**) patient survival.

As shown in Fig. 3A, overall graft survival was significantly impaired with decreasing serum bilirubin levels (*P* < 0.001). The 10-year graft survival rates were 91.5% in the highest quartile (Q4), 89.8% in the second quartile (Q3), 84.5% in the third quartile (Q2), and 76.8% in the lowest quartile (Q1; *P* < 0.001). Death-censored graft survival was also significantly different among the groups (Fig. 3B). Recipients with high bilirubin levels had the best death-censored graft survival, whereas recipients with low bilirubin levels had the worst death-censored graft survival. Compared with the highest bilirubin group, the lowest and third quartile groups were significantly associated with graft loss and death-censored graft failure (Table 4). The risks for graft loss and death-censored graft failure in the lowest bilirubin group remained significant, although attenuated, in the fully adjusted model (hazard ratio [HR] 2.64; 95% confidence interval [CI] 1.67–4.18; and HR 2.96; 95% CI 1.62–5.40, respectively).

**Table 4:** Association between serum bilirubin levels and graft loss, death-censored graft failure, and mortality.

Variables	Model 1		Model 2		Model 3	
	HR (95% CI)	*P*	HR (95% CI)	*P*	HR (95% CI)	*P*
Graft loss						
Bilirubin group (4th quartile)	1 [Reference]		1 [Reference]		1 [Reference]	
1st quartile	3.37 (2.16–5.26)	< 0.001	2.97 (1.89–4.67)	< 0.001	2.64 (1.67–4.18)	< 0.001
2nd quartile	2.06 (1.27–3.34)	0.003	1.82 (1.12–2.97)	0.016	1.74 (1.07–2.84)	0.026
Death-censored graft failure						
Bilirubin group (4th quartile)	1 [Reference]		1 [Reference]		1 [Reference]	
1st quartile	3.81 (2.12–6.84)	< 0.001	3.51 (1.94–6.36)	< 0.001	2.97 (1.63–5.42)	< 0.001
2nd quartile	2.24 (1.19–4.24)	0.013	2.03 (1.07–3.85)	0.029	1.87 (0.99–3.56)	0.055
All-cause mortality						
Bilirubin group (4th quartile)	1 [Reference]		1 [Reference]		1 [Reference]	
1st quartile	2.64 (1.32–5.27)	0.006	2.17 (1.07–4.37)	0.031	2.07 (1.01–4.22)	0.047
2nd quartile	1.64 (0.77–3.50)	0.204	1.37 (0.64–2.95)	0.417	1.40 (0.65–3.00)	0.399

Model 1 adjusted for age, sex, and BMI.

Model 2 adjusted for age, sex, BMI, donor status (deceased versus living), HLA mismatch, donor age, donor eGFR, and donor sex.

Model 3 adjusted for age, sex, BMI, donor status (deceased versus living), HLA mismatch, donor age, donor eGFR, donor sex, dialysis duration, and eGFR.

During follow-up, 66 (4.1%) patients died, including 34 who died of infectious causes, 12 who died of malignancies, 7 who died of cardiovascular causes, and 13 who died of other causes. Compared with the highest bilirubin group, the lowest bilirubin group was significantly associated with all-cause mortality (Fig. 3C). A multivariable Cox regression confirmed that the lowest serum bilirubin group was independently associated with higher mortality (HR, 2.07; 95% CI, 1.01–4.22).

### Serum bilirubin levels and graft rejection

**Figure 4:**
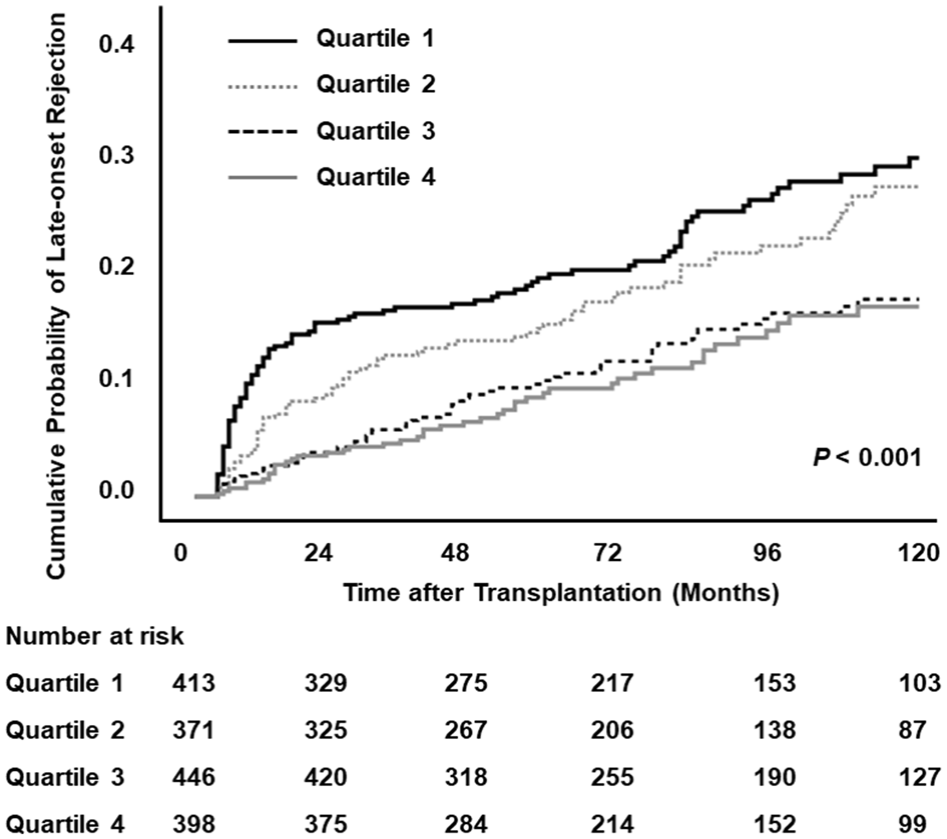
Late-onset rejection according to serum bilirubin level.

During the follow-up period, 769 rejection episodes occurred in 286 patients (294 early-onset and 475 late-onset rejection episodes). The incidences of early-onset rejection (within 3 months from transplant) in Q1, Q2, Q3, and Q4, were 12.1%, 7.3%, 6.1%, and 4.0%, respectively (*P* < 0.001). The ten-year cumulative probabilities for late-onset rejection (> 3 months after transplant), in Q1, Q2, Q3, and Q4, were 28.6%, 26.0%, 17.1%, and 16.4%, respectively (Fig. 4, *P* < 0.001). A greater proportion of patients in the Q1 and Q2 groups (9.4% and 9.7%, respectively) experienced multiple late-onset rejection episodes compared with those in the Q3 and Q4 groups (6.1% and 4.0%, respectively; *P* < 0.001).

### Serum bilirubin and graft renal function

**Figure 5:**
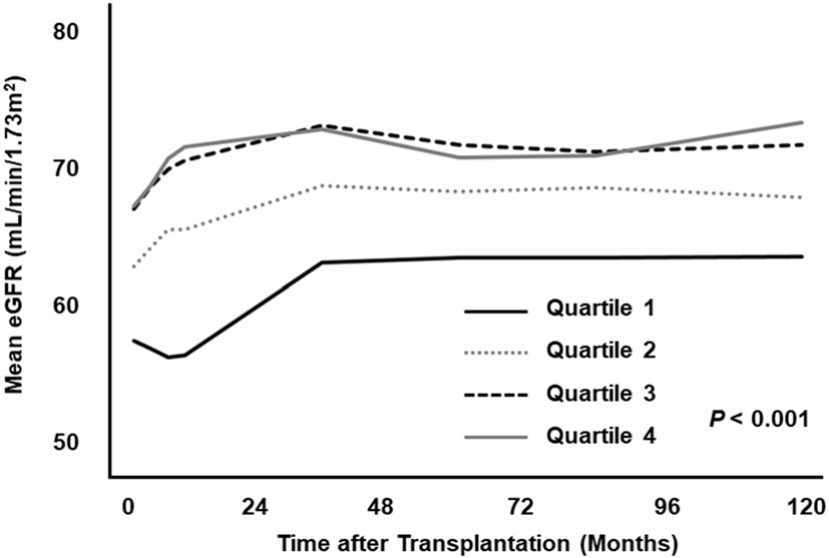
Changes in graft function according to serum bilirubin level.

The mean estimated glomerular filtration rate (eGFR) was consistently lower in the lowest bilirubin (Q1) and third quartile groups (Q2) than in the highest bilirubin (Q4) and second quartile groups (Q3), throughout the follow-up period (Fig. 5). The mean eGFRs at 5-years post-transplantation were 63.5 ± 22.5, 68.2 ± 21.0, 71.4 ± 19.7, and 70.5 ± 18.7 mL/min/1.73 m^2^, respectively (*P* < 0.001). The significant effects of serum bilirubin on graft function were maintained, regardless of donor status (data not shown).

## Discussion

In this study, we found that lower serum bilirubin levels were associated with higher risks of graft loss, death-censored graft failure, and all-cause mortality, after adjustment for graft function, donor status, human leukocyte antigen (HLA) mismatch, and transplant characteristics. Furthermore, patients with lower serum bilirubin levels were at increased risk of rejection and exhibited consistently lower eGFR values compared with those with higher serum bilirubin levels.

Bilirubin, a breakdown product of heme catabolism, has long been regarded as a useless and toxic metabolite. However, growing evidence has shown that bilirubin exerts antioxidant, anti-inflammatory, and lipid peroxidation inhibitory effects^12,13,16^. Large epidemiological studies have shown that mildly elevated bilirubin levels are associated with decreased risks of cardiovascular and metabolic diseases^8,9,17^. In addition, animal experiments and clinical studies have found that bilirubin can prevents the progression of renal disease, which is characterized by increased oxidative stress, inflammatory activation, and metabolic disturbances^11^. These previous findings suggested that serum bilirubin levels might be associated with graft outcomes after kidney transplantation^15^.

The causes of graft failure are heterogeneous and multifactorial^3,4^. Non-immunological injuries, including oxidative stress, cardiovascular and metabolic disturbances, and endothelial dysfunction, can significantly contribute to graft failure. Despite the introduction of potent immunosuppression, graft rejection and alloimmune injury continue to play important roles in graft failure^18^. As mentioned above, accumulating evidence has shown that bilirubin exerts a protective role against renal disease progression, through various mechanisms^10,11^. Bilirubin also has an immunomodulatory effect, which is expected to reduce graft rejection after kidney transplantation^14,15^. However, scarce data exist regarding the association between serum bilirubin levels and kidney transplant outcomes^19^.

Our findings suggested that serum levels of bilirubin are inversely associated with graft and patient survival. Previous studies have assessed the association between serum bilirubin levels and graft outcomes in kidney transplant recipients^19,20^. However, they used a single serum bilirubin measurement, taken at one time point after transplantation, and more patients received cyclosporine in these studies, in contrast with the current clinical practice of tacrolimus administration. In addition, the range of time between transplantation and enrollment was wide (IQR, 2.6–11.4 years)^19^. Compared with previous studies, we measured serum bilirubin levels several times after transplantation, within the same time period (3–12 months) for all patients, and confirmed the low intra-individual variability in serum bilirubin levels. Cardiovascular disease-related mortality was found to be significantly lower in this study cohort. The high proportion of living donor transplants, the relatively young ages of included patients, and the reduced prevalence of cardiovascular risk factors may explain the low cardiovascular disease-related mortality among our cohort. Thus, our findings suggested a potential beneficial effect of circulating bilirubin for kidney transplant patients, beyond cardiovascular protection.

Despite significant advances in short-term outcomes after kidney transplantation, long-term outcomes have not improved substantially^1,2^. The impact of late-onset rejections on long-term graft outcomes has been increasingly recognized as immunologic assays and diagnostic criteria have improved^18^. We found that low serum bilirubin levels were associated with late-onset rejection. In addition, significantly more patients with low bilirubin levels experienced multiple rejection episodes than those with higher bilirubin levels. Bilirubin has immunomodulatory activities that can reduce effector T-cell responses, intercept the complement cascade, and promote the expansion of the regulatory T-cell population, which may play beneficial roles in the prevention of graft rejection^15,21,22^. In addition, bilirubin inhibits the cell-surface expression of the major histocompatibility complex (MHC) II class molecules B7 and CD28^23,24^. Animal model studies have shown that bilirubin can induce tolerance after transplantation^25^. Lee et al*.* also found that lower serum bilirubin levels 1 year after transplantation were associated with increased graft rejection^20^.

To the best of our knowledge, this study represents the largest cohort study conducted to evaluate the effects of serum bilirubin on transplant outcomes after kidney transplantation. Although determining causality is beyond the scope of this study, multiple mechanisms have been proposed to explain the beneficial effects of bilirubin^9,11,14^. To date, the insolubility of bilirubin has limited its clinical application, but several therapeutic approaches are continuously being developed^26,27^. The potential beneficial effects on bilirubin indicate that bilirubin could represent a valuable target for pharmacologic interventions designed to improve long-term transplant outcomes^9,27–29^.

Our study has several limitations. First, this study was performed as a retrospective study at a single institution. However, this study design allowed us to ascertain granular data and maintain homogeneity during the follow-up protocol and the application of immunosuppressive regimens. Second, this study included a large number of living donor transplant patients with low cardiovascular risks; thus, our results might have limited generalizability. We may have underestimated the beneficial effects of circulating bilirubin on the transplant outcomes associated with cardiovascular protection. We also noted an imbalance of donor characteristics among groups. Although the impact of serum bilirubin on the transplant outcomes remained significant in the fully adjusted model including donor characteristics, the results of this study should be interpreted cautiously. Third, the lack of fractionation of total bilirubin into conjugated and unconjugated fractions limited the ability to evaluate which bilirubin fraction was associated with graft outcomes. Finally, as with any observational study, we can neither prove causality nor exclude the possibility of potential confounders.

In conclusion, we found that serum bilirubin levels were inversely associated with graft and patient survival in kidney transplant recipients. Patients with lower serum bilirubin levels were also associated with increased risks of late-onset rejection and inferior graft function.

## Methods

### Study population

A total of 2044 adult patients (aged ≥ 18 years) underwent kidney transplantation at the Severance Hospital, Seoul, Republic of Korea, between January 2003 and December 2017. Patients who underwent multi-organ transplantations and HLA-incompatible kidney transplant recipients were excluded. We also excluded patients who experienced graft loss within 3 months, who were diagnosed with viral hepatitis, and who lacked sufficient data. After the exclusion of ineligible patients, 1628 kidney transplant recipients were enrolled in this study. Patients were grouped into sex-specific quartiles, based on mean serum bilirubin levels assessed between 3 and 12 months post-transplantation (Fig. 1).

### Immunosuppression

Immunosuppression was performed as described previously^30^. Most patients received induction immunosuppression with basiliximab or anti-thymocyte globulin. Maintenance immunosuppression consisted of calcineurin inhibitors (tacrolimus or cyclosporine), prednisolone, and mycophenolate mofetil (MMF). The initial tacrolimus dosage was administered orally, at 0.1 mg/kg, twice daily. Subsequent doses were adjusted to maintain a target trough concentration between 5 and 8 ng/mL. The initial oral dosage of cyclosporine was 5 mg/kg twice daily, and it was adjusted to achieve a trough level of 100–200 ng/mL. The initial dose of methylprednisolone (500–1000 mg) was gradually reduced to oral prednisolone (5–10 mg/day) during the first 3 weeks after transplantation. The initial dose of MMF was 1.0 g/day, which was subsequently adjusted to minimize adverse events, such as gastrointestinal trouble or neutropenia.

### Clinical and laboratory measurements

Routine biochemical tests, including the assessment of total serum bilirubin, were performed every month for the first post-transplantation year, and every 3 months thereafter. Serum total bilirubin was measured using an absorptiometric assay with chemical oxidation by vanadic acid on an automatic analyzer. GFR was evaluated using the Chronic Kidney Disease Epidemiology Collaboration Eq. ^31^.

Renal biopsies were performed in cases of acute allograft dysfunction (> 30% increase in serum creatinine levels compared with baseline or proteinuria > 500 mg/day). Allograft biopsy samples were processed using light, immunofluorescent, and electron microscopy at the time of biopsy. All acute rejections were confirmed by biopsy and classified according to the most recent Banff criteria at the time of biopsy^32^.

### Definition and study endpoints

Graft loss was defined as the return to long-term dialysis, re-transplantation, or death with a functioning graft^33^. Graft survival was calculated from the date of transplantation to the date of graft loss or December 31, 2019 (the end of the follow-up period). In cases of death with a functioning graft, graft survival was censored at the time of death. Patient survival was evaluated as the date of transplantation to the date of death or the last follow-up. Late-onset rejection was defined as any biopsy-confirmed rejection that occurred more than 3 months post-transplantation.

The primary study endpoint was graft loss. Secondary endpoints included death-censored graft failure, all-cause patient mortality, late-onset rejection, and graft function.

### Statistical analysis

Data were expressed as the frequency, the mean and standard deviation, or the median and interquartile range, depending on the data type. Chi-square or Fisher’s exact tests were used as appropriate to compare categorical variables. Continuous variables were compared using one‐way analysis of variance, for parametric data, or the Kruskal–Wallis test, for nonparametric data. Multivariable logistic regression was performed using the lowest bilirubin group as an outcome variable. Covariates included baseline characteristics and laboratory findings at 3 months post-transplantation. Graft and patient survival rates were analyzed using Kaplan–Meier curves and the log-rank test. Cox proportional hazard regression models were used to evaluate the associations between serum bilirubin levels and time to graft loss, death-censored graft failure, and all-cause mortality. Initial models were adjusted, a priori, for serum bilirubin level, age, sex, and body mass index. Extended models were further adjusted, a priori, for donor status (deceased versus living), HLA mismatch, donor age, and donor sex. Fully adjusted models were a priori adjusted for dialysis duration and eGFR. Statistical analyses were performed using SPSS software (version 25.0; SPSS Inc., Chicago, IL, USA), and *P* < 0.05 was considered significant.

### Ethics statement

All study procedures were conducted in accordance with the Declaration of Helsinki and were approved by the Institutional Review Board of Severance Hospital (2020–1309-001). Informed consent was waived by the Institutional Review Board of Severance Hospital because of the study’s retrospective design.

## Data Availability

The datasets generated during and/or analyzed during the current study are available from the corresponding author on reasonable request.
